# Precipitation effects on grassland plant performance are lessened by hay harvest

**DOI:** 10.1038/s41598-022-06961-7

**Published:** 2022-02-28

**Authors:** Karen Castillioni, Michael A. Patten, Lara Souza

**Affiliations:** 1 Oklahoma Biological Survey and Department of Microbiology and Plant Biology, University of Oklahoma, Norman, OK 73019 USA; 2 Department of Ecology, Evolution and Behavior, University of Minnesota, Saint Paul, MN 55108 USA; 3 Ecology Research Group, Faculty of Biosciences and Aquaculture, Nord University, 7729 Steinkjer, Norway

**Keywords:** Biodiversity, Climate-change ecology, Community ecology, Plant ecology, Abiotic, Biotic, Drought, Flooding, Heat, Herbivory

## Abstract

Climate and human management, such as hay harvest, shape grasslands. With both disturbances co-occurring, understanding how these ecosystems respond to these combined drivers may aid in projecting future changes in grasslands. We used an experimental precipitation gradient combined with mimicked acute hay harvest (clipping once a year) to examine (1) whether hay harvest influences precipitation effects on plant performance (cover and height) and (2) the role of inter-specific responses in influencing plant performance. We found that hay harvest reduced the strength of precipitation effects on plant performance through changes in bare-ground soil cover. Species performance were mainly influenced by change in abiotic factors, often responding negatively, as hay harvest increased bare-ground amount. Conversely, altered precipitation without hay harvest promoted plant species performance through abiotic factors change first, followed by biotic. Most species, including the dominant grass *Schizachyrium scoparium*, increased their performance with greater leaf area index (proxy for canopy structure). Our experiment demonstrates that plant performance responds directly to abiotic factors with hay harvest, but indirectly without hay harvest. Positive effects of increasing precipitation were likely due to microhabitat amelioration and resource acquisition, thus inclusion of hay harvest as a disturbance lessens positive impacts of biotic variables on species performance to climate change.

## Introduction

Climate and human management are some of the important factors that shape vegetation dynamics in grasslands. Climate—in particular factors that influence temperature and soil moisture—is the primary determinant of plant productivity, with human management operating within constraints imposed by moisture availability^1,2^. Climate models forecast increased precipitation variability in grasslands^3^, leading to more frequent dry periods in many regions^4,5^. Altered precipitation already has created novel abiotic and biotic conditions across ecosystems, resulting in community shifts that alter ecosystem structure and function^6,7^. Combining altered precipitation and hay harvest, studies suggest that their interactions could substantially affect plant community composition and total aboveground net primary productivity (ANPP)^8–10^. Because these drivers may influence community and ecosystem responses differently^11^, they must be manipulated and studied in combination to draw realistic conclusions about overall plant performance under future environmental change scenarios^12^.

Soil moisture availability limits net primary production in grasslands, with growing-season precipitation determining ANPP over time^13,14^. Like ANPP, grassland species richness often increases with precipitation^15^, while species might undergo abundance change (species re‐ordering^6^). Increased drought incidence will therefore negatively impact ANPP in grasslands but have a variable impact on plant community composition. Abiotic stressors caused by altered precipitation drive community change, yet biotic structure (e.g., biomass production, canopy structure and community richness) influences community‐level responses by mediating effects of these stressors^16^. Species in a community might ameliorate the environmental stress for other species by facilitating their coexistence, establishment or growth^17–19^. For example, neighboring species ameliorate some or many stressful environmental conditions, causing positive impacts on focal species^20^, and plants of different growth forms can alter the canopy structure of plant communities^21^, resulting in competitive hierarchies with effects on the plant performance due to the directional supply of light^22–24^. Plant communities thus exhibit a particular suite of varied species performance as a result of particular combinations of biotic structure^25,26^, yet under severe environmental change, biotic structure may become unimportant to determine plant performance, relative to the effect of the abiotic stress. Only the most stress-tolerant species can persist under harsh conditions^27–29^.

In the US Great Plains, hay harvest is a common human management practice that acts as a strong driver of plant community structure and ecosystem function^10^. Hay harvest, whether acute or chronic^30^, increases ground-level light penetration and surface temperature, which can have mixed impacts on plants^12^. Above-ground biomass removal by hay harvest can be beneficial for growth of early emerging species due to reduced physical barrier for growth and light limitation^31^. Alternatively, hay harvest increases soil insolation, resulting in higher surface temperatures, ultimately filtering for heat tolerant species^32^. Plants surrounded by soil exposure experience greater rates of attack from herbivores because of greater plant apparency^33^. Further, increased bare-ground may increase visibility to herbivores but may also expose plants to greater drought stress^34^. Increased bare-ground amount also reduces densities of plant neighbors, which can be facilitators in harsh conditions^34^. Combined, drought and hay harvest may result in higher surface temperatures and reduced moisture—by less precipitation or more water loss via soil evaporation^35^—than found with altered precipitation or hay harvest alone, reducing both plant growth and cover.

Plant communities dominated by different functional types could differ in their response to abiotic or biotic factors that ultimately shape their performance (cover and height). Plant functional traits may drive the structure of biological communities^36–38^. Functional traits that allow tolerance to drought may overlap with traits that provide tolerance to disturbance like vegetation clipping, thus traits can ultimately determine the response of functional types^39,40^. For example, C_4_ plants use water more efficiently^41^, and some are adapted to disturbances such as grazing, which should give them higher competitive ability to handle water stress and defoliation relative to C_3_ counterparts^42,43^. Research focusing on responses of C_3_ and C_4_ species must consider adaptations of these functional types to tease apart how performance of each is shaped by abiotic vs. biotic variables across an environmental gradient.

Understanding the role of interactive effects of disturbances is important for modelling and projecting future plant community dynamics and the stability of ecosystem functions as climate changes. Here, we report results from a novel field experiment in which we manipulated precipitation at multiple levels with rain-out shelters—a gradient of increasing precipitation (from extreme drought [− 100% precipitation] to precipitation addition [+ 50% precipitation])—and tested acute clipping once a year (hereafter hay harvest). We tested for hay harvest as an acute disturbance (i.e., occurring once a year) as we were not aiming to address the effects of its frequency, but occurrence. We define hay harvest as a disruption of biotic structure that leads to a pulse in available resources, such as light and space^30^. We examined the effects of altered precipitation in two scenarios, with and without acute hay harvest, to address the following questions: (1) Can hay harvest influence the effect of a gradient from drought to increasing precipitation on abiotic and biotic conditions, and consequently alter overall plant performance?; (2) What is the influence of inter-specific responses in driving plant performance responses to hay harvest and a gradient from drought to increasing precipitation? We hypothesized that (1) hay harvest will lessen the effects of increasing precipitation by reducing plant cover and resulting in decreased plant performance (i.e., height and cover)^44^; and that (2) differences in inter-specific responses (via inter-specific differences in functional traits) will play a key role in determining plant performance under hay harvest and increasing precipitation, as plant species have varied tolerance to soil moisture and clipping disturbance^39,40^.

## Methods

### Study site

We studied the plant species and community responses in 2017 from June to August at Kessler Atmospheric and Ecological Field Station (KAEFS), a mesic and mixed-grass prairie in central Oklahoma, USA (34° 59′ N, 97° 31′ W), last farmed > 45 years ago. Permission to use this study site was obtained from KAEFS Steering Committee. The study site is dominated by C_4_ and C_3_ graminoids, and C_3_ forbs^43^. Annual precipitation in 2017 was 992.12 mm (historical average in 1998–2016: 872.76 mm) and mean air temperature was 16.66 °C (historical average in 1998–2016: 16.15 °C) (Supplementary Fig. S1, Oklahoma Climatological Survey).

### Experimental design

To determine the response of focal plants to a precipitation gradient and clipping, we used replicated rain-out shelters established in January/February 2016 to create multiple levels of precipitation. This experimental study is part of Drought-Net, a coordinated global network examining terrestrial ecosystem sensitivity to drought. We used a randomized block split-plot design with seven precipitation treatments (five water exclusion levels [− 20%, − 40%, − 60%, − 80%, and − 100% of the ambient precipitation], one water addition [+ 50% of the ambient precipitation], and a control [0% change in precipitation or no change]) replicated three times (replication number follows Drought-Net protocol) for a total of 21, 2 × 2 m plots (Supplementary Fig. S2). Subplots are 1 × 1 m plots within the 21 2 × 2 m plots. One of the trade-offs to the low replication in our experimental design is the wider spectrum of treatment levels we used which allowed us to explore differing precipitation scenarios^45^. Soil moisture reflected the proposed precipitation gradient^12^. In addition, one subplot within each precipitation treatment plot was clipped once to mimic hay harvest at the end of the growing season in September 2016. All aboveground biomass was clipped at a height of 10 cm from ground level and removed from the subplot to mimic hay harvest^46^. Diagonally from the clipping subplot was the unclipped control subplot.

### Plant performance

To determine the effects of the precipitation gradient and hay harvest on the species performance—quantified by plant height and plant cover—we selected the nine most common plant species (focal plants: six C_3_ species—i.e., five forbs and one graminoid—and three C_4_ grasses) at our study site. The selected species and their mean (± SE henceforth) relative plant cover were estimated in 2016 (baseline year): the C_3_ forbs are *Ambrosia psilostachya* (7.3 ± 1.1%), *Erigeron strigosus* (1.7 ± 0.5%), *Croton monanthogynus* (2.7 ± 0.5%), *Solidago nemoralis* (0.1 ± 0.1%), *and Symphyotrichum ericoides* (3.7 ± 0.8%); while the C_3_ graminoid is *Dichanthelium oligosanthes* (4.6 ± 0.8%), and C_4_ graminoids are *Sorghastrum nutans* (5.1 ± 0.8%), *Sporobolus compositus* (5.3 ± 1.0%) and *Schizachyrium scoparium* (37.2 ± 2.1%). These species were also selected because they occurred in 70% of the plots. We tagged one adult individual of each species in each experimental plot, i.e., clipped and unclipped subplots across the precipitation treatments. For each individual tagged species, we estimated percentage foliar cover (i.e., vegetative cover including stems and leaves) as a measure of cover using a modified Braun–Blanquet cover-abundance scale that included seven categories of percentage foliar cover: 1%, 1–5%, 5–25%, 25–50%, 50–75%, 75–95%, 95–100%^47^. We used the median of each assigned cover class as the cover for each individual tagged species in a plot, and maximum percentage foliar cover between June and July sampling periods for each species. We measured height by holding the tallest leaf upright from the base of the stem to the tip of the leaf once in early August 2017. Our study complies with the IUCN Policy Statement on Research Involving Species at Risk of Extinction and the Convention on the Trade in Endangered Species of Wild Fauna and Flora.

### Biotic variables

To determine the effects of a precipitation gradient and hay harvest on biotic variables, we measured community richness as the total number of species in each plot once in the peak of the growing season in July 2017. We estimated ANPP at the end of the growing season (September 2017) by using clipping standing biomass in clipped subplots (cut at 10 cm from ground level in 1 × 1 m subplots). Standing biomass for ANPP from unclipped plots was clipped in 20 × 100 cm strips also in September, following Drought-Net protocol, and scaled up to g m^−2^ as a control for clipped subplots. Clipped materials were oven-dried and weighed. We measured leaf area index (LAI)—canopy structure based on the projected area of leaves—averaged across the months of June, July and August 2017 by using AccuPAR LP-80.

### Abiotic variables

To determine the effects of a precipitation gradient and hay harvest on abiotic variables, we measured soil moisture, soil temperature and bare-ground cover^12^. Soil probes (Decagon 5TM, ICT International) continuously measured percentage volumetric water content (VWC, i.e., soil moisture, Supplementary Table S1) and soil temperature (°C) at a depth of 10 cm, every 10 min, from May 2017 to September 2017, in each clipped and unclipped subplot nested in precipitation treatment plots. We then averaged soil moisture and soil temperature within the same time frame, corresponding to the plant growing season. Additionally, we visually estimated bare-ground cover (%) using the same modified Braun–Blanquette cover-abundance scale.

### Statistical analysis

We used a piecewise structural equation model (SEM)^48,49^ that accounted for both direct and indirect effects to achieve a system-understanding of the major drivers of plant performance. A similar approach has been used to pinpoint the direct and indirect effects of our precipitation gradient experiment and clipping on arthropod abundance and diversity in our previous study^12^. Structural equation modelling is particularly useful in large-scale correlative studies because it allows us to partition causal influences among multiple variables, and to separate the direct and indirect effects of the predictors included in the model^50^. Our a priori model based on our current knowledge is available in Figure S3. We built two piecewise SEMs, one for altered precipitation effects *with hay harvest* and another for *without hay harvest*. All piecewise SEMs contained plant cover and height of all focal species of the community as the response variable, with soil moisture, soil temperature, and bare-ground cover as abiotic predictor variables, and community richness, ANPP, and LAI as biotic predictor variables. Separate SEMs for C_3_ forbs, C_3_ graminoid and C_4_ graminoids were also performed. Before running SEMs, we used *Z*-scores to scale variables. We included species identity as a random factor in our models because individual responses can influence overall plant focal height and cover. In order to resolve pseudo-replication due to repeated sampling, we also included plot nested within block as a random variable in all mixed model regressions. We used tests of directed separation to include missing paths. We used a single piecewise SEM model based on our a priori model for altered precipitation effects under hay harvest and no hay harvest and did not remove non-significant links. In comparison with traditional SEM, piecewise SEMs are less restricted by the number of links per sample size, and Fisher’s *C* is used as the goodness-of-fit statistic^48,49^. As in traditional SEM, a non-significant *P*-value indicates a well-fit model. We conducted Piecewise SEMs by using piecewiseSEM^49^ and nlme^51^ packages in R ^52^.

We used generalized linear mixed models (GLMM) to test the significance of individual relationships on variables (i.e., height and cover) for each species. Plot was used as a random effect nested within block. The level of significance for all statistical tests was α = 0.05. A gamma error distribution (inverse link) was used to model continuous variables, such as species-specific height and cover, as well as abiotic and biotic variables; while Poisson error distribution (log link) was used to model discrete counting variables, such as community richness when we assessed precipitation and hay harvest effects. To test the independent effects of the precipitation gradient and hay harvest on biotic and biotic variables, we conducted a GLMM with the same approach described above. We log-transformed response variables to better meet normality assumptions. All models were checked for overdispersion and normal distribution. We performed models by using the glmer function in the lme4 package in R^52^.

## Results

### Precipitation gradient and hay harvest effects on overall plant performance

Effects of increased precipitation on plant performance were lessened with vs. without hay harvest. A precipitation gradient without hay harvest increased plant performance through changes in both abiotic and biotic conditions. In SEMs with and without hay harvest, changes in focal plant height were correlated positively with changes in focal plant cover.

**Figure 1:**
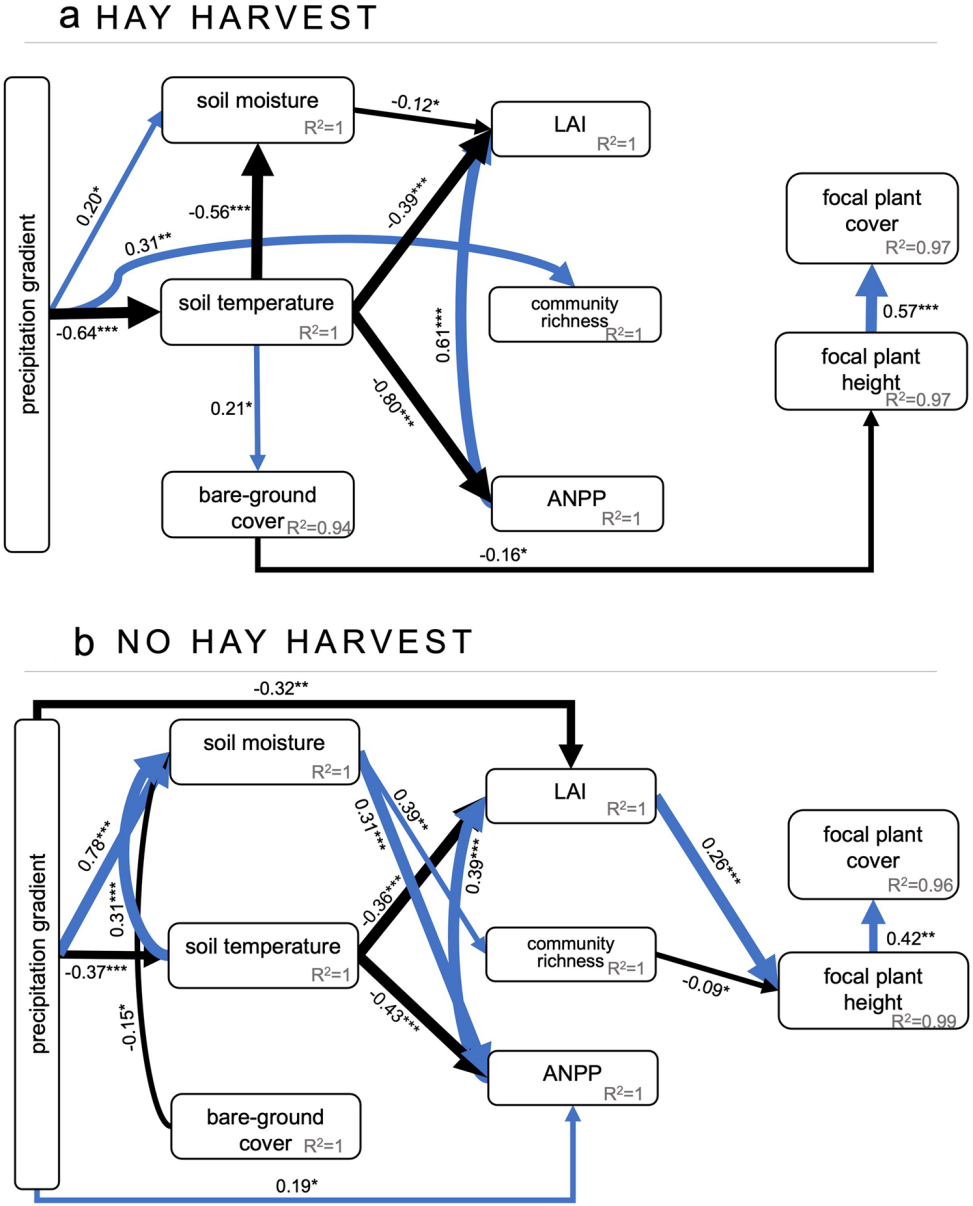
Piecewise Structural Equation Models (SEMs) describing the relationships among plant performance (focal plant cover, focal plant height), biotic variables (LAI—leaf area index, ANPP—aboveground net primary productivity, community richness), abiotic variables (soil moisture, soil temperature, bare-ground cover) in plots with hay harvest (**a**) no hay harvest (**b**) across precipitation treatments. Conditional R^2^ values (i.e., including fixed and random effects) are under each predicted variable and standardized path estimates are provided next to each path with line thickness scaled based on the strength of the relationship (see “Methods” for variable descriptions): *P ≤ 0.05, **P ≤ 0.01, ***P ≤ 0.001. Only significant relationships (*P* ≤ 0.05) are shown. Blue and black arrows indicate positive and negative relationships, respectively. Arrow widths are proportional to the strength of the relationship. The proportion of variance explained (R^2^) appears alongside the response variable in the model. Model estimates, standard errors, and *P*-values for significant and non-significant relationships are provided in Supplementary Tables S2–S3.

Hay harvest had a strong negative effect on bare-ground cover (*P* =  < 0.001, Supplementary Table S4): bare ground increased from 4.2 ± 0.85% in *no* hay harvested plots to 21.0 ± 1.70% in hay harvested plots. In the SEM, bare-ground cover increased with soil temperature, which decreased in response to increasing precipitation (Fig. 1a). This change in bare-ground cover was the only significant link to focal plant performance (plant height, regression coefficient: − 0.16) in the Hay Harvest SEM (Fig. 1a, Supplementary Table S2, Fisher’s C = 5.15, AICc = 151.15, *P* = 0.52). Under hay harvest, increasing precipitation directly promoted community richness (regression coefficient: 0.31) and soil moisture (regression coefficient: 0.19). In turn, decrease in soil temperature (through precipitation increase) was negatively correlated with LAI (regression coefficient: − 0.11) and ANPP (regression coefficient: − 0.80), although none of these changes affected plant performance.

In the No Hay Harvest SEM, increasing precipitation strongly influenced plant performance through three routes: (1) increasing precipitation increased soil moisture that increased community richness but, subsequently, decreased overall focal plant height (regression coefficient: − 0.09) (Fig. 1b, Supplementary Table S3, Fisher’s C = 12.03, AICc = 158.04, *P* = 0.06); (2) increasing precipitation was directly and negatively associated to LAI (regression coefficient: − 0.32), and to soil temperature, which decreased LAI but increased plant performance; (3) increasing precipitation was directly and positively associated with increase in ANPP (regression coefficient: 0.19) and soil moisture (regression coefficient: 0.78)—which also increased ANPP (regression coefficient: 0.31)—subsequently, promoting LAI (regression coefficient: 0.39) and plant performance.

### Species identity influence on plant performance

We further examined direct relationships between key biotic and abiotic variables that promoted change in plant performance variables for each species using GLMMs. These analyses allowed us to explore how focal species identity could influence overall performance in our SEMs.

**Figure 2:**
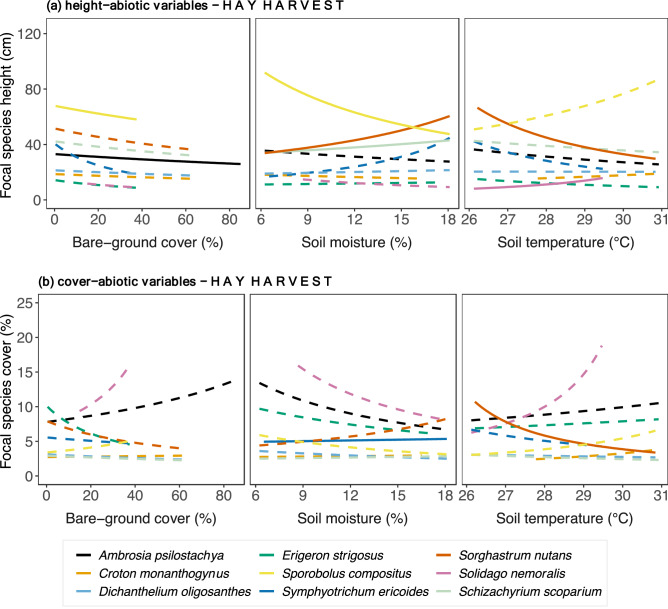
Focal species relationships between (**a**) height, (**b**) cover and abiotic variables in plots with *hay harvest* across the precipitation gradient. Relationships were estimated by fitting Generalized Linear Mixed Models with log link to both species-specific height and abiotic variables (soil temperature, soil moisture, bare-ground cover). Continuous lines indicate significant relationships, while dashed lines indicate non-significant. P-values are shown in Supplementary Table S4.

**Figure 3:**
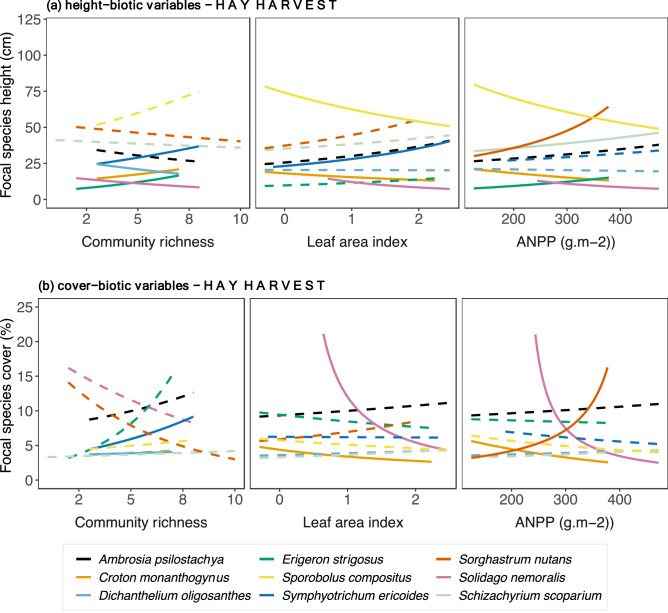
Focal species relationships between (**a**) height, (**b**) cover and biotic variables relationships in plots with *hay harvest* across the precipitation gradient. Relationships were estimated by fitting Generalized Linear Mixed Models with log link to both species-specific height and biotic variables (community richness, leaf area index, above-ground net primary). Continuous lines indicate significant relationships, while dashed lines indicate non-significant. *P*-values are shown in Table 1.
  Bare-ground cover predominantly correlated negatively to focal species height, whereas soil moisture and soil temperature shared a mix of positive and negative relationships across species (Fig. 4a and Supplementary Table S5). Height of the forbs *Ambrosia psilostachya* and *Symphyotrichum ericoides* and the grasses *Dichanthelium oligosanthes* and *Sporobolus compositus* correlated negatively with bare-ground cover. Increased soil moisture corresponded to increased height of the forbs *Croton monanthogynus* and *Solidago nemoralis* and the C_4_ grass *Sorghastrum nutans* but to decreased height of the forbs *Ambrosia psilostachya* and *Erigeron. strigosus*. Height correlated positively with soil temperature in the forbs *Dichanthelium oligosanthes*, *Solidago nemoralis,* and *Erigeron strigosus,* and the C_4_ grass *Sporobolus compositus* but negatively with *Croton monanthogynus* and *Sorghastrum nutans.* Among focal species (Fig. 4b, Supplementary Table S6), *Ambrosia psilostachya* cover correlated positively withes in bare-ground cover but negatively to *Sporobolus compositus* cover. Soil moisture correlated positively with cover of only one species, *Solidago nemoralis*. High values of soil temperature correlated positively with cover of *Dichantelium oligosanthes* and negatively with cover of *Ambrosia psilostachya*.

- Hay harvest across the precipitation gradient
  In this scenario, bare-ground cover had direct negative effects on species performance in the Hay Harvest SEM. *Ambrosia psilostachya* (C_3_ forb) and *Sporobolus compositus* (C_4_ graminoid) height decreased with increased bare-ground cover (Fig. 2a, Supplementary Table S5). Other abiotic variables shared positive and negative relationships with plant performance variables (Fig. 2a, Supplementary Table S5). For example, only C_4_ graminoids responded to changes in soil moisture—*Schizachyrium scoparium* and *Sorghastrum nutans* height increased with increased soil moisture, while *Sporobolus compositus* height was negatively related to soil moisture. Only two species’ heights changed with increased temperature—*Sorghastrum nutans* was negatively affected, but *Solidago nemoralis* increased. Regarding focal species cover (Fig. 2b, Supplementary Table S6), the C_3_ *Symphyotrichum ericoides* slightly increased with soil moisture; in contrast, the C_4_ *Sorghastrum nutans* decreased with increased soil temperature.
  Only *Solidago nemoralis* and *Dichanthelium oligosanthes* height decreased with increased community richness, but height of *Croton monanthogynus, Erigeron strigosus, Symphyotrichum ericoides* increased (Fig. 3). None of the C_4_ species responded (Fig. 3). High values of LAI corresponded to increased height of *Symphyotrichum. ericoides*, but the opposite was held for *Croton monanthogynus*, *Solidago. nemoralis* and *Sporobolus compositus* (Fig. 3a). Increased ANPP was associated with increased height of the *Schizachyrium scoparium* and *Sorghastrum nutans*, and with *Erigeron strigosus* but decreased height of *Croton monanthogynus*, *Solidago nemoralis* and *Sporobolus compositus* (Fig. 3a). Regarding focal species cover (Fig. 3b, Table 2), the cover of C_3_ grass *Dichanthelium oligosanthes* and the C_3_ forb *Symphyotrichum ericoides* correlated positively with community richness. Increased LAI corresponded to decreased cover of *Croton monanthogynus* and *Solidago nemoralis*. The same pattern held for ANPP, except that *Sorghastrum nutans* cover increased with increased ANPP.
- No hay harvest across the precipitation gradient

**Figure 4:**
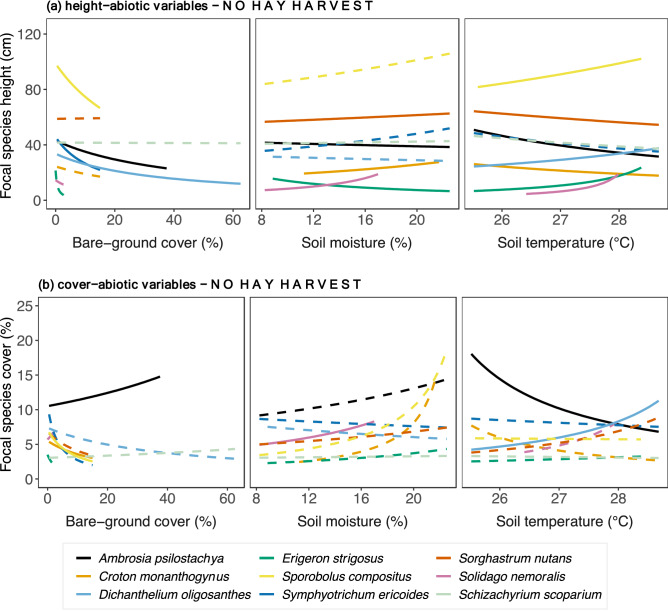
Focal species relationships between (**a**) height, (**b**) cover and abiotic variables relationships in plots with *no hay harvest* across the precipitation gradient. Relationships were estimated by fitting Generalized Linear Mixed Models with log link to both species-specific height and abiotic variables (soil temperature, soil moisture, bare-ground cover). Continuous lines indicate significant relationships, while dashed lines indicate non-significant. *P*-values are shown in Supplementary Table S5.

**Figure 5:**
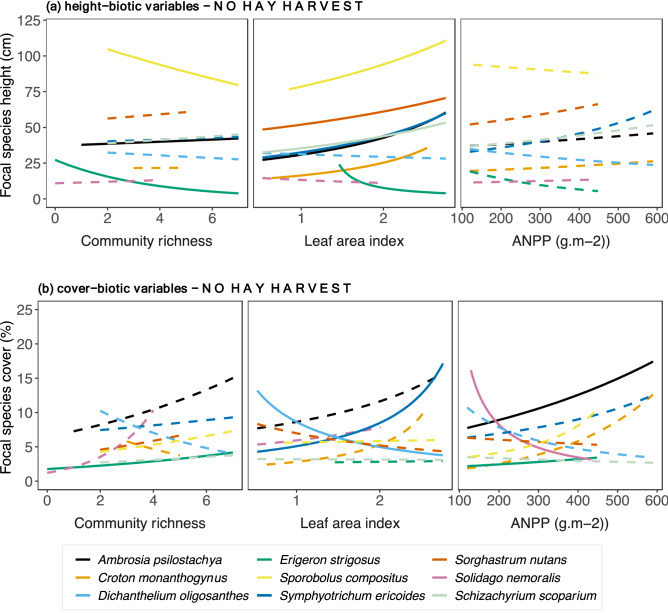
Focal species relationships between (**a**) height, (**b**) cover and biotic variables relationships in plots with *no hay harvest* across the precipitation gradient. Relationships were estimated by fitting Generalized Linear Mixed Models with log link to both species-specific cover and biotic variables (community richness, leaf area index, above-ground net primary productivity). Continuous lines indicate significant relationships, while dashed lines indicate non-significant. *P*-values are shown in Table 2.

The precipitation gradient and abiotic conditions altered biotic variables, such as ANPP, community richness and LAI, subsequently influencing focal species performance (Fig. 1). Height of *Ambrosia psilostachya*, a C_3_ forb, correlated positively with community richness, while height of *Erigeron strigosus* and *Sporobolus compositus* decreased with increased community richness. Height of the C_3_ forbs *Ambrosia psilostachya, Croton monanthogynus*, and *Symphyotrichum ericoides*) and all C_4_ graminoid species increased with increased LAI (Fig. 5a and Table 1) but was uncorrelated to ANPP. Among focal species (Fig. 5b, Table 2), *Erigeron strigosus* cover was the only one positively associated to community richness. Higher values of LAI correlated positively with *Symphyotrichum ericoides* cover but negatively *Dichanthelium oligosanthes* cover. Among forb, *Ambrosia psilostachya* and *Erigeron strigosus* cover increased with increased ANPP, while *Solidago nemoralis* cover decreased*.*

**Table 1:** GLMM of main effects of plant richness, leaf area index and ANPP on focal species *height*, under *hay harvest* vs. *no hay harvest* across the precipitation gradient. Significant *P* (≤ 0.05) shown in bold.

Species	Community richness		Leaf area index		ANPP (g m^−2^)	
	Chisq	*P*	Chisq	*P*	Chisq	*P*
***Ambrosia psilostachya***						
Hay harvest	3.11	0.08	0.97	0.32	0.03	0.87
No hay harvest	2566.5	**< 0.001**	21.33	**< 0.001**	0.91	0.34
***Croton monanthogynus***						
Hay harvest	4.12	**0.04**	61,680	**< 0.001**	0.64	**< 0.01**
No hay harvest	0.34	0.56	5.13	**0.02**	< 0.01	0.96
***Erigeron strigosus***						
Hay harvest	61,202	**< 0.001**	2.87	0.09	9.08	**< 0.01**
No hay harvest	5.43	**0.02**	318,861	**< 0.001**	2.40	0.12
***Solidago nemoralis***						
Hay harvest	347.7	**< 0.001**	34.12	**< 0.001**	8.11	**< 0.01**
No hay harvest	< 0.01	0.97	0.96	0.33	0.11	0.74
***Symphyotrichum ericoides***						
Hay harvest	4.66	**0.03**	309,944	**< 0.001**	0.85	0.36
No hay harvest	0.02	0.88	2262.2	**< 0.001**	0.11	0.74
***Dichanthelium oligosanthes***						
Hay harvest	2582.7	**< 0.001**	0.09	0.76	0.06	0.80
No hay harvest	0.54	0.46	< 0.01	0.93	2.14	0.14
***Schizachyrium scoparium***						
Hay harvest	0.21	0.65	2.32	0.13	9.61	**< 0.01**
No hay harvest	0.81	0.37	17,077	**< 0.001**	1.53	0.22
***Sorghastrum nutans***						
Hay harvest	0.01	0.91	2.85	0.09	56.87	**< 0.001**
No hay harvest	0.60	0.44	5.31	**0.02**	1.25	0.26
***Sporobolus compositus***						
Hay harvest	2.58	0.10	3.91	**0.05**	4.35	**0.04**
No hay harvest	3.68	**0.05**	8.96	**< 0.01**	0.622	0.43

**Table 2:** GLMM of main effects of plant richness, leaf area index and ANPP on focal species *cover*, under *hay harvest* vs. *no hay* harvest across the precipitation gradient. Significant *P* (≤ 0.05) shown in bold.

Species	Community richness		Leaf area index		ANPP (g m^−2^)	
	Chisq	*P*	Chisq	*P*	Chisq	*P*
***Ambrosia psilostachya***						
Hay harvest	0.32	0.57	0.04	0.84	0.29	0.59
No hay harvest	0.76	0.38	1.04	0.30	16.91	**< 0.001**
***Croton monanthogynus***						
Hay harvest	0.07	0.78	5.17	**0.02**	46,174,087	**< 0.001**
No hay harvest	0.11	0.74	5.54	0.11	0.69	0.40
***Erigeron strigosus***						
Hay harvest	3.40	0.06	0.09	0.76	0.97	0.32
No hay harvest	6.92	**< 0.01**	0.02	0.90	51.99	**< 0.001**
***Solidago nemoralis***						
Hay harvest	1.57	0.21	13.69	**< 0.001**	52,869	**< 0.001**
No hay harvest	2.36	0.12	0	0.99	40.82	**< 0.001**
***Symphyotrichum ericoides***						
Hay harvest	6436.9	**< 0.001**	0.09	0.75	< 0.01	0.93
No hay harvest	0.01	0.93	64,687	**< 0.001**	0.49	0.48
***Dichanthelium oligosanthes***						
Hay harvest	< 0.01	0.98	436.51	**< 0.001**	0.16	0.69
No hay harvest	0.49	0.49	4.70	**0.3**	3.19	0.07
***Schizachyrium scoparium***						
Hay harvest	< 0.01	0.98	2.52	0.11	0.04	0.84
No hay harvest	0.03	0.86	< 0.01	0.93	0.99	0.32
***Sorghastrum nutans***						
Hay harvest	0.81	0.36	1.27	0.26	3.80	**0.05**
No hay harvest	< 0.01	0.93	0.45	0.50	0.10	0.74
***Sporobolus compositus***						
Hay harvest	0.02	0.89	0.11	0.74	0.49	0.48
No hay harvest	0.04	0.85	0.70	0.40	0.92	0.34

### Precipitation gradient and hay harvest effects on functional groups’ plant performance

- C_3_ forbs: Hay harvest influenced C_3_ forbs’ plant performance mainly through biotic change (Supplementary Tables S7–S8), irrespective of treatment. Increasing precipitation affected plant performance by increasing soil moisture, which increased LAI and, subsequently, plant height (regression coefficient: 0.67). We found the same pattern for increased precipitation without hay harvest (regression coefficient: 0.36). In both SEMs, focal plant height was positively correlated to plant abundance.
- C_3_ graminoid: Neither the precipitation gradient nor hay harvest affected *Dichanthelium oligosanthes* performance (Supplementary Tables S9–S10).
- C_4_ graminoids: Focal C_4_ graminoid plant performance mirrored overall plant performance, with exceptions only when precipitation change occurred without hay harvest (Supplementary Tables S11–S12). In that SEM, LAI was the only biotic variable correlated with focal plant height but not with community richness. In both SEMs, focal plant height was positively correlated with plant cover.

## Discussion

### Precipitation gradient and hay harvest effects on overall plant performance

We provide new insights, from a novel experiment design, that acute hay harvest reduces the effect of a precipitation gradient on plant performance. A key abiotic variable, bare-ground soil cover, mediated precipitation effects on plant performance. Specifically, increases in bare-ground cover, due to vegetation removal by hay harvest, directly hindered plant height. Compared to precipitation change without hay harvest, the effect of hay harvest changes the drivers of plant performance from being abiotic alone to a combination of biotic plus abiotic. The piecewise structural equation modelling (SEM) allowed us to identify the most important ecological predictors as well as the associations between precipitation change, abiotic variables and biotic variables as drivers of plant performance (plant height and cover) in hay harvest vs. no hay harvest conditions. Our experimental results demonstrate how hay harvest influences the trajectory of altered precipitation on plant performance. This finding is especially important as current climate change predictions for temperate grasslands include increased precipitation variability, which will co-occur with human management.

Bare-ground was an important abiotic driver of plant performance of the community. The amount of bare-ground surrounding individual plants can expose them to greater UV radiation, increase drought stress, and reduce densities of plant neighbors which can ameliorate harsh conditions^34,44^. Moving forward, disentangling the relative importance of bare-ground cover and pathways leading to plant performance will require the expansion of experimental and descriptive approaches, for example, measurements incorporating other abiotic conditions or resource availability. Measurement of plant traits and abilities associated with resource uptake, competition, and drought tolerance may shed light on the reasons for bare-ground increase with altered precipitation with hay harvest^37,53,54^.

Biotic variables were the main drivers of plant performance with altered precipitation only (under no hay harvest). Increase in richness was related to the increase in soil moisture along the precipitation gradient, allowing more species to coexist. Higher number of plant species likely increased competitive interactions, hindering target plant performance^55^. In contrast, increase in soil moisture also promoted LAI through increase in ANPP, having a positive effect on overall plant performance. Higher values of LAI likely was positively associated with greater overall plant performance^7–19^. Thus, biotic variables directly influenced by abiotic conditions and resources, ultimately affected plant performance^16,55–57^. Additionally, net biotic interactions around focal species, the relative frequency and intensity of facilitative (positive) and competitive (negative) interactions between plants, are assumed to change temporally, becoming more positive under increasing drought stress and more negative as drought stress decreases^56^. Conversely, increased precipitation affects the rate of resource acquisition, specifically water, altering vegetation density and the intensity and importance of net biotic interactions, all of which will influence drought induced compositional and performance changes^56^.

We also found that plant height predicted foliar cover; they covaried positively in our models. Plant stature is associated with the ability to intercept light from neighbors, thus shading competitors^37^. In contrast, immediate changes in foliar cover are limited by a trade-off between tall plants with long leaves, and short plants with many leaves^38^. This means that plant growth in height is an important variable influencing foliar lateral spread for light interception and interaction with neighboring plants.

### Species identity influence on plant performance

We further explored responses of plant species in relation to biotic and abiotic variables to better understand the role of species identity in driving overall plant responses. We found that responses were species-specific as hypothesized. Our previous study^43^ on species-specific responses to precipitation and clipping showed a small number of significant interactive effects between these treatments. Here we found that most species performance metrics, but especially plant height, mainly were influenced by change in abiotic variables; if altered precipitation co-occurs with hay harvest, it negatively impacts plant performance. In this context, only height of C_4_ graminoids responded positively to greater soil moisture. As a result, the dominant grass *Schizachyrium scoparium* and subdominant grass *Sorghastrum nutans*, responded positively to increases in soil moisture, suggesting water limitation in this ecosystem^58^. Cover of only two species (a forb and a grass) were associated with greater soil moisture and soil temperature change, highlighting the importance of changes in height to define plant performance when precipitation change is concurrent with hay harvest. Finally, although not statistically significant in the SEM models, we found a tendency for mixed positive and negative relationships between plant performance and biotic variables in the context of precipitation change and hay harvest.

In contrast, most species increased in performance with higher values of LAI when precipitation occurred alone. A total of six out of nine species were mainly influenced by increases in LAI, including all C_4_ graminoids. These results show that these species are benefited by greater LAI and increased community richness when only precipitation increased; yet are not influenced by biotic variables if hay harvest co-occurs with changes in precipitation. Neighbors around focal plants ameliorate some or many environmental conditions, allowing species to grow despite harsh conditions^20^. Hence, we posit that microhabitat amelioration by neighbors’ presence was key for other species performance (*Ambrosia psilostachya*, *Croton monanthogynus*, *Symphyotrichum ericoides, Schizachyrium scoparium, Sorghastrum nutans* and *Sporobolus compositus*), including species that are known to be less abundant in the community. Thus, vegetation removal by harvest disrupts the positive effects of biotic structure when grassland species undergo changes in precipitation.

### Precipitation gradient and hay harvest effects on functional groups plant performance

Plant performance, both of individuals and specifically of focal C_4_ graminoids, was influenced similarly, a finding that suggests C_4_ graminoids determined overall plant performance. The SEM for C_3_ grasses showed no significant change of plant performance, but C_3_ grasses were only represented by a single species (*Dichanthelium oligosanthes*). These results are expected because C_4_ graminoids are the dominant functional group in our study site. By contrast, C_3_ forbs performance mainly increased through biotic change, independently of the precipitation manipulation. However, the positive effect of biotic variables (ANPP, LAI and community richness) was slightly stronger with hay harvest when considering C_3_ species. This positive effect potentially allowed more plant growth, thus likely generating more light and space for growth conditions for C_3_ species^59^.

## Conclusions

We demonstrate the role of interactive effects of disturbances in shaping plant performance. Hay harvest lessens precipitation effects on biotic and abiotic variables to influence plant performance. We further conclude that abiotic factors (i.e., soil temperature and soil moisture) and biotic factors (i.e., ANPP and LAI) are important drivers of plant performance along a precipitation gradient. Abiotic factors often drive response to climate drivers at the larger scale, whereas biotic factors at the local scale^60^. However, this effect will depend on the type of occurring disturbances. Our integrative disturbance approach can be extended to test the generality of adaptation to changes in abiotic and indirect biotic factors in other plant groups and in other regions with different precipitation conditions, like arid and moist environments. It is also important to study other metrics of plant performance to further understand the impacts of climate change and human management. Finally, more broadly, pathway analysis approaches applied to a variety of systems and questions in climate change ecology is an important means through which we can explain the changes of biodiversity.

## Supplementary Information

Supplementary Information.

## Data Availability

Dataset is available on SHAREOK University of Oklahoma Libraries (https://shareok.org/handle/11244/334592).
