# Supplementary material for: Precipitation effects on grassland plant performance are lessened by hay harvest
Source: Sci Rep. 2022 Feb 28;12:3282. doi: 10.1038/s41598-022-06961-7 (PMC8885915; doi:10.1038/s41598-022-06961-7)
Supplement: Supplementary file 1 — Supplementary Information. [file 41598_2022_6961_MOESM1_ESM.docx]

*Scientific Reports*: Research Article

Supporting Information

Precipitation effects on grassland plant performance are lessened by hay harvest

**Author names and addresses:**

Karen Castillioni (KC)^1,2*^, Michael A. Patten (MAP)^3^, Lara Souza (LS)^1^

**1:** Oklahoma Biological Survey and Department of Microbiology and Plant Biology, University of Oklahoma, Norman, Oklahoma, 73019, USA

**2:** Department of Ecology, Evolution and Behavior, University of Minnesota, Saint Paul, MN, 55108, USA

**3:** Ecology Research Group, Faculty of Biosciences and Aquaculture, Nord University, N-7729 Steinkjer, Norway

**Corresponding author*:**

Karen Castillioni

Email: casti311@umn.edu

**ORCID identifiers:** **Karen Castillioni**: 0000-0002-9205-7631; **Michael A. Patten**: 0000-0001-9689-2645; **Lara Souza**: 0000-0001-6005-866

**Table S1.** Mean and standard error (SE) for soil moisture (%) in each precipitation level *with* and *without* *hay harvest* during the growing season May-September 2017.

| **Precipitation level** | **Hay harvest** | **Mean** | **SE** |
| --- | --- | --- | --- |
| -100 | yes | 12.88 | 0.44 |
| -80 | yes | 15.07 | 0.41 |
| -60 | yes | 16.93 | 0.48 |
| -40 | yes | 13.92 | 0.45 |
| -20 | yes | 15.18 | 0.44 |
| 0 | yes | 17.72 | 0.49 |
| 50 | yes | 17.97 | 0.35 |
| -100 | no | 14.67 | 0.42 |
| -80 | no | 16.96 | 0.41 |
| -60 | no | 14.57 | 0.35 |
| -40 | no | 16.07 | 0.42 |
| -20 | no | 16.62 | 0.44 |
| 0 | no | 14.00 | 0.37 |
| 50 | no | 21.50 | 0.31 |

**Table S2.** Model estimates, standard errors (SE), and p-values (*P*) from *hay harvest* under altered precipitation piecewise Structural Equation Model (Fig. 2a) depicting the direct and indirect effects of precipitation treatments and clipping on Focal species height and foliar cover. Significant *P* (≤0.05) values are shown in bold.

| **Response** | **Predictor** | **Estimate** | **SE** | ***P*** |
| --- | --- | --- | --- | --- |
| Focal plant height | Richness | 0.03 | 0.06 | 0.60 |
| Focal plant height | ANPP | -0.16 | 0.14 | 0.23 |
| Focal plant height | LAI | 0.08 | 0.15 | 0.59 |
| Focal plant height | Soil temperature | -0.22 | 0.13 | 0.10 |
| Focal plant height | Soil moisture | -0.09 | 0.09 | 0.29 |
| Focal plant height | Bare-ground cover | -0.16 | 0.06 | **0.01** |
| Focal plant height | Precipitation treatment | -0.08 | 0.08 | 0.31 |
| Focal plant abundance | Focal plant height | 0.57 | 0.11 | **<0.001** |
| Focal plant abundance | Richness | 0.06 | 0.08 | 0.46 |
| Focal plant abundance | ANPP | 0.12 | 0.17 | 0.49 |
| Focal plant abundance | LAI | -0.06 | 0.19 | 0.76 |
| Focal plant abundance | Soil temperature | 0.01 | 0.17 | 0.97 |
| Focal plant abundance | Soil moisture | -0.07 | 0.11 | 0.48 |
| Focal plant abundance | Bare-ground cover | 0.11 | 0.08 | 0.17 |
| Focal plant abundance | Precipitation treatment | -0.05 | 0.10 | 0.61 |
| Richness | Soil temperature | 0.20 | 0.13 | 0.15 |
| Richness | Soil moisture | 0.07 | 0.12 | 0.57 |
| Richness | Bare-ground cover | 0.03 | 0.09 | 0.75 |
| Richness | Precipitation treatment | 0.31 | 0.12 | **<0.01** |
| LAI | ANPP | 0.62 | 0.06 | **<0.001** |
| LAI | Soil temperature | -0.39 | 0.07 | **<0.001** |
| LAI | Soil moisture | -0.12 | 0.05 | **0.02** |
| LAI | Bare-ground cover | -0.05 | 0.04 | 0.14 |
| LAI | Precipitation treatment | 0.03 | 0.05 | 0.50 |
| ANPP | Soil temperature | -0.80 | 0.08 | **<0.001** |
| ANPP | Soil moisture | -0.13 | 0.08 | 0.09 |
| ANPP | Bare-ground cover | -0.10 | 0.06 | 0.09 |
| ANPP | Precipitation treatment | 0.10 | 0.07 | 0.18 |
| Bare-ground cover | Soil temperature | 0.21 | 0.11 | **0.01** |
| Soil temperature | Precipitation treatment | -0.64 | 0.07 | **<0.001** |
| Soil moisture | Bare-ground cover | -0.05 | 0.06 | 0.42 |
| Soil moisture | Soil temperature | -0.56 | 0.08 | **<0.001** |
| Soil moisture | Precipitation treatment | 0.20 | 0.08 | **0.02** |

**Table S3.** Model estimates, standard errors (SE), and p-values (*P*) from *without hay harvest* under altered precipitation piecewise Structural Equation Model (Fig. 2b) depicting the direct and indirect effects of precipitation treatments and clipping on Focal species height and foliar cover. Significant *P* (≤0.05) values are shown in bold.

| **Response** | **Predictor** | **Estimate** | **SE** | ***P*** |
| --- | --- | --- | --- | --- |
| Focal plant height | Richness | -0.09 | 0.04 | **0.04** |
| Focal plant height | ANPP | -0.06 | 0.06 | 0.31 |
| Focal plant height | LAI | 0.26 | 0.05 | **<0.001** |
| Focal plant height | Soil temperature | 0.07 | 0.06 | 0.20 |
| Focal plant height | Soil moisture | 0.04 | 0.06 | 0.47 |
| Focal plant height | Bare-ground cover | -0.07 | 0.04 | 0.10 |
| Focal plant height | Precipitation treatment | 0.06 | 0.06 | 0.36 |
| Focal plant abundance | Focal plant height | 0.41 | 0.14 | **<0.01** |
| Focal plant abundance | Richness | 0.08 | 0.09 | 0.35 |
| Focal plant abundance | ANPP | 0.08 | 0.12 | 0.49 |
| Focal plant abundance | LAI | -0.09 | 0.11 | 0.42 |
| Focal plant abundance | Soil temperature | -0.04 | 0.11 | 0.70 |
| Focal plant abundance | Soil moisture | 0.17 | 0.12 | 0.17 |
| Focal plant abundance | Bare-ground cover | 0.00 | 0.09 | 0.97 |
| Focal plant abundance | Precipitation treatment | -0.15 | 0.13 | 0.25 |
| Richness | Soil temperature | -0.18 | 0.10 | 0.07 |
| Richness | Soil moisture | 0.39 | 0.12 | **<0.01** |
| Richness | Bare-ground cover | 0.06 | 0.09 | 0.52 |
| Richness | Precipitation treatment | -0.12 | 0.13 | 0.37 |
| LAI | ANPP | 0.40 | 0.10 | **<0.001** |
| LAI | Soil temperature | -0.36 | 0.10 | **<0.001** |
| LAI | Soil moisture | 0.11 | 0.11 | 0.32 |
| LAI | Bare-ground cover | -0.14 | 0.08 | 0.06 |
| LAI | Precipitation treatment | -0.32 | 0.11 | **<0.01** |
| ANPP | Soil temperature | -0.43 | 0.07 | **<0.001** |
| ANPP | Soil moisture | 0.31 | 0.08 | **<0.001** |
| ANPP | Bare-ground cover | -0.09 | 0.06 | 0.14 |
| ANPP | Precipitation treatment | 0.19 | 0.09 | **0.04** |
| Bare-ground cover | Soil temperature | 0.12 | 0.09 | 0.18 |
| Soil temperature | Precipitation treatment | -0.37 | 0.08 | **<0.001** |
| Soil moisture | Bare-ground cover | -0.15 | 0.06 | **0.03** |
| Soil moisture | Soil temperature | 0.31 | 0.07 | **<0.001** |
| Soil moisture | Precipitation treatment | 0.78 | 0.07 | **<0.001** |

**Table S4.** GLMMs of main effects of the precipitation gradient and hay harvest on biotic variables (community richness, LAI, ANPP) and abiotic variables (soil moisture, soil temperature and bare-ground cover). Significant *P* (≤0.05) shown in bold.

|  | **Precipitation gradient** | | **Hay harvest** | |
| --- | --- | --- | --- | --- |
| **Species** | **Chisq** | ***P*** | **Chisq** | ***P*** |
| **Biotic variables** |  |  |  |  |
| community richness | 6.60 | **0.01** | 16.78 | **<0.001** |
| LAI | 0.63 | 0.43 | 26.11 | **<0.001** |
| ANPP | 4.45 | **0.03** | <0.01 | 0.94 |
| **Abiotic variables** |  |  |  |  |
| soil moisture | 6.59 | **0.01** | 3.80 | **0.05** |
| soil temperature | 8.36 | **<0.01** | 29.65 | **<0.001** |
| bare-ground cover | <0.01 | 0.99 | 149.35 | **<0.001** |

**Table S5.** GLMMs of main effects of bare-ground cover, soil moisture and soil temperature on focal species-specific ***height***, *under hay harvest* vs. *no hay harvest*. Significant *P* (≤0.05) shown in bold.

|  | **Bare-ground cover (%)** | | **Soil moisture (%)** | | **Soil temperature (°C)** | |
| --- | --- | --- | --- | --- | --- | --- |
| **Species** | **Chisq** | ***P*** | **Chisq** | ***P*** | **Chisq** | ***P*** |
| *Ambrosia psilostachya* |  |  |  |  |  |  |
| hay harvest | 56.54 | **<0.001** | 1.90 | 0.17 | 0.07 | 0.79 |
| no hay harvest | 6.35 | **0.01** | 4574298 | **<0.001** | 116057 | **<0.001** |
| *Croton monanthogynus* |  |  |  |  |  |  |
| hay harvest | 0.31 | 0.58 | 1.90 | 0.17 | 0.45 | 0.50 |
| no hay harvest | <0.01 | 0.96 | 4574298 | **<0.001** | 78480 | **<0.001** |
| *Erigeron strigosus* |  |  |  |  |  |  |
| hay harvest | 0.47 | 0.49 | 0.67 | 0.41 | 1.16 | 0.28 |
| no hay harvest | 1.86 | 0.17 | 19784692 | **< 0.001** | 1123909 | **<0.001** |
| *Solidago nemoralis* |  |  |  |  |  |  |
| hay harvest | 0.14 | 0.70 | 0.67 | 0.41 | 47659 | **<0.001** |
| no hay harvest | 1.76 | 0.18 | 49310885 | **< 0.001** | 33.13 | **<0.001** |
| *Symphyotrichum ericoides* |  |  |  |  |  |  |
| hay harvest | 1.75 | 0.19 | 0.67 | 0.41 | 2.62 | 0.10 |
| no hay harvest | 5.32 | **0.02** | 0.98 | 0.32 | 2.77 | 0.09 |
| *Dichanthelium oligosanthes* |  |  |  |  |  |  |
| hay harvest | 0.07 | 0.79 | 0.14 | 0.71 | <0.01 | 0.93 |
| no hay harvest | 1393.4 | **< 0.001** | <0.001 | 0.98 | 4.98 | **0.02** |
| *Schizachyrium scoparium* |  |  |  |  |  |  |
| hay harvest | 2.67 | 0.10 | 3752530 | **< 0.001** | 0.13 | 0.72 |
| no hay harvest | 0.16 | 0.69 | 0.02 | 0.89 | 0.74 | 0.39 |
| *Sorghastrum nutans* |  |  |  |  |  |  |
| hay harvest | 1.18 | 0.28 | 7.39 | **<0.01** | 10.37 | **<0.01** |
| no hay harvest | 0.16 | 0.69 | 1010023 | **< 0.001** | 82.15 | **<0.001** |
| *Sporobolus compositus* |  |  |  |  |  |  |
| hay harvest | 8.83 | **<0.01** | 5.38 | **0.02** | 2.62 | 0.10 |
| no hay harvest | 16.07 | **< 0.001** | 1.45 | 0.23 | 6175.6 | **<0.001** |

**Table S6.** GLMMs of main effects of bare-ground cover, soil moisture and soil temperature on focal species-specific ***abundance***, *under hay harvest* vs. *no hay harvest*. Significant *P* (≤0.05) shown in bold.

|  | **Bare-ground cover (%)** | | **Soil moisture (%)** | | **Soil temperature (°C)** | |
| --- | --- | --- | --- | --- | --- | --- |
| **Species** | **Chisq** | ***P*** | **Chisq** | ***P*** | **Chisq** | ***P*** |
| *Ambrosia psilostachya* |  |  |  |  |  |  |
| hay harvest | 1.47 | 0.22 | 0.8 | 0.78 | 0.12 | 0.72 |
| no hay harvest | 4844.4 | **< 0.001** | 0.25 | 0.61 | 4.63 | **0.03** |
| *Croton monanthogynus* |  |  |  |  |  |  |
| hay harvest | 0.002 | 0.96 | <0.001 | 0.97 | 1.26 | 0.26 |
| no hay harvest | 433009 | **<0.001** | 0.36 | 0.55 | 2.46 | 0.12 |
| *Erigeron strigosus* |  |  |  |  |  |  |
| hay harvest | 0.34 | 0.55 | <0.01 | 0.93 | 0.35 | 0.55 |
| no hay harvest | 0 | 0.99 | 0.36 | 0.55 | 0.27 | 0.60 |
| *Solidago nemoralis* |  |  |  |  |  |  |
| hay harvest | 0.45 | 0.50 | 0.05 | 0.81 | 2.97 | 0.8 |
| no hay harvest | 0.44 | 0.50 | 50414290 | **<0.001** | 76876 | **<0.001** |
| *Symphyotrichum ericoides* |  |  |  |  |  |  |
| hay harvest | 2.37 | 0.12 | 12832559 | **<0.001** | 0.42 | 0.51 |
| no hay harvest | 3.57 | 0.06 | 0.27 | 0.60 | 0.28 | 0.60 |
| *Dichanthelium oligosanthes* |  |  |  |  |  |  |
| hay harvest | 0.16 | 0.69 | 1.10 | 0.29 | 0.08 | 0.78 |
| no hay harvest | 2.19 | 0.14 | 0.12 | 0.73 | <0.001 | **<0.001** |
| *Schizachyrium scoparium* |  |  |  |  |  |  |
| hay harvest | 0.69 | 0.41 | 0.04 | 0.84 | 0.39 | 0.53 |
| no hay harvest | 3.24 | 0.07 | 0.16 | 0.69 | 0.06 | 0.80 |
| *Sorghastrum nutans* |  |  |  |  |  |  |
| hay harvest | 0.53 | 0.46 | 0.78 | 0.38 | 4.72 | **0.03** |
| no hay harvest | 0.16 | 0.68 | 0.14 | 0.70 | 0.18 | 0.67 |
| *Sporobolus compositus* |  |  |  |  |  |  |
| hay harvest | 0.36 | 0.55 | 1.76 | 0.18 | 0.38 | 0.53 |
| no hay harvest | 14.17 | **<0.001** | 0.99 | 0.32 | 0.06 | 0.80 |

**Table S7.** Model estimates, standard errors (SE), and p-values (*P*) from *hay harvest* under altered precipitation piecewise Structural Equation Model depicting the direct and indirect effects of precipitation treatments and clipping on Focal species height and foliar cover in **C_3_ forbs**. Significant *P* (≤0.05) values are shown in bold. SEM AIC = 155.52, F=25.52 and *P*-value = 0.27.

| **Response** | **Predictor** | **Estimate** | **SE** | ***P*** |
| --- | --- | --- | --- | --- |
| Focal plant height | Bare-ground cover | -0.14 | 0.12 | 0.23 |
| Focal plant height | Community richness | 0.08 | 0.11 | 0.47 |
| Focal plant height | ANPP | -0.46 | 0.23 | 0.05 |
| Focal plant height | LAI | 0.67 | 0.24 | **<0.01** |
| Focal plant height | Soil moisture | 0.03 | 0.13 | 0.83 |
| Focal plant height | Precipitation treatment | -0.18 | 0.14 | 0.19 |
| Focal plant abundance | Precipitation treatment | -0.11 | 0.11 | 0.29 |
| Focal plant abundance | Focal plant height | 0.46 | 0.13 | **<0.001** |
| Community richness | Soil temperature | 0.08 | 0.18 | 0.66 |
| Community richness | Soil moisture | 0.26 | 0.18 | 0.16 |
| Community richness | Bare-ground cover | 0.05 | 0.13 | 0.71 |
| LAI | Community richness | -0.05 | 0.05 | 0.33 |
| LAI | ANPP | 0.56 | 0.09 | **<0.001** |
| LAI | Soil temperature | -0.40 | 0.11 | **<0.001** |
| LAI | Soil moisture | -0.11 | 0.07 | 0.16 |
| LAI | Bare-ground cover | -0.10 | 0.05 | 0.08 |
| LAI | Precipitation treatment | 0.06 | 0.07 | 0.36 |
| ANPP | Community richness | -0.13 | 0.08 | 0.11 |
| ANPP | Soil temperature | -0.86 | 0.11 | **<0.001** |
| ANPP | Soil moisture | -0.09 | 0.11 | 0.43 |
| Bare-ground cover | Soil temperature | 0.23 | 0.12 | 0.06 |
| Soil temperature | Precipitation treatment | -0.67 | 0.10 | **<0.001** |
| Soil moisture | Bare-ground cover | -0.02 | 0.10 | 0.86 |
| Soil moisture | Soil temperature | -0.62 | 0.13 | **<0.001** |
| Soil moisture | Precipitation treatment | 0.12 | 0.12 | 0.33 |

**Table S8.** Model estimates, standard errors (SE), and p-values (*P*) from *without hay harvest* under altered precipitation piecewise Structural Equation Model depicting the direct and indirect effects of precipitation treatments and clipping on Focal species height and foliar cover **C_3_ forbs**. Significant *P* (≤0.05) values are shown in bold. SEM AIC = 160.76, F= 30.76 and *P*-value = 0.10.

| **Response** | **Predictor** | **Estimate** | **SE** | ***P*** |
| --- | --- | --- | --- | --- |
| Focal plant height | Bare-ground cover | -0.11 | 0.08 | 0.20 |
| Focal plant height | Community richness | -0.13 | 0.09 | 0.14 |
| Focal plant height | ANPP | -0.10 | 0.10 | 0.33 |
| Focal plant height | LAI | 0.36 | 0.09 | **<0.001** |
| Focal plant height | Soil moisture | 0.23 | 0.10 | 0.02 |
| Focal plant height | Precipitation treatment | -0.11 | 0.10 | 0.30 |
| Focal plant abundance | Precipitation treatment | 0.13 | 0.10 | 0.21 |
| Focal plant abundance | Focal plant height | 0.57 | 0.12 | **<0.001** |
| Community richness | Soil temperature | -0.22 | 0.12 | 0.08 |
| Community richness | Soil moisture | 0.43 | 0.11 | **<0.001** |
| Community richness | Bare-ground cover | 0.20 | 0.12 | 0.11 |
| LAI | Community richness | 0.22 | 0.12 | 0.07 |
| LAI | ANPP | 0.43 | 0.14 | **<0.01** |
| LAI | Soil temperature | -0.31 | 0.14 | **0.03** |
| LAI | Soil moisture | -0.04 | 0.17 | 0.82 |
| LAI | Bare-ground cover | -0.17 | 0.12 | 0.15 |
| LAI | Precipitation treatment | -0.27 | 0.16 | 0.09 |
| ANPP | Community richness | -0.24 | 0.11 | **0.03** |
| ANPP | Soil temperature | -0.52 | 0.09 | **<0.001** |
| ANPP | Soil moisture | 0.58 | 0.10 | **<0.001** |
| Bare-ground cover | Soil temperature | 0.36 | 0.12 | **<0.01** |
| Soil temperature | Precipitation treatment | -0.35 | 0.12 | **<0.01** |
| Soil moisture | Bare-ground cover | -0.13 | 0.11 | 0.21 |
| Soil moisture | Soil temperature | 0.33 | 0.11 | **<0.01** |
| Soil moisture | Precipitation treatment | 0.78 | 0.10 | **<0.001** |

**Table S9.** Model estimates, standard errors (SE), and p-values (*P*) from *hay harvest* under altered precipitation piecewise Structural Equation Model depicting the direct and indirect effects of precipitation treatments and clipping on Focal species height and foliar cover in **C_3_ graminoid**. Significant *P* (≤0.05) values are shown in bold. SEM AIC = 153.50, F=7.50 and *P*-value = 0.27.

| **Response** | **Predictor** | **Estimate** | **SE** | ***P*** |
| --- | --- | --- | --- | --- |
| Focal plant height | Richness | -0.39 | 0.41 | 0.36 |
| Focal plant height | ANPP | -0.15 | 0.92 | 0.87 |
| Focal plant height | LAI | 0.67 | 0.90 | 0.48 |
| Focal plant height | Soil temperature | 0.57 | 0.78 | 0.48 |
| Focal plant height | Soil moisture | 0.48 | 0.52 | 0.39 |
| Focal plant height | Bare-ground cover | -0.46 | 0.38 | 0.26 |
| Focal plant height | Precipitation treatment | -0.41 | 0.46 | 0.40 |
| Focal plant abundance | Focal plant height | 0.59 | 0.26 | 0.06 |
| Focal plant abundance | Community richness | 0.12 | 0.32 | 0.71 |
| Focal plant abundance | ANPP | 0.26 | 0.68 | 0.71 |
| Focal plant abundance | LAI | 0.02 | 0.69 | 0.98 |
| Focal plant abundance | Soil temperature | -0.45 | 0.60 | 0.48 |
| Focal plant abundance | Soil moisture | -0.67 | 0.41 | 0.15 |
| Focal plant abundance | Bare-ground cover | -0.14 | 0.31 | 0.66 |
| Focal plant abundance | Precipitation treatment | -0.16 | 0.36 | 0.66 |
| Community richness | Soil temperature | 0.15 | 0.39 | 0.71 |
| Community richness | Soil moisture | -0.32 | 0.43 | 0.47 |
| Community richness | Bare-ground cover | -0.30 | 0.27 | 0.30 |
| Community richness | Precipitation treatment | 0.38 | 0.38 | 0.34 |
| LAI | ANPP | 0.75 | 0.19 | **<0.01** |
| LAI | Soil temperature | -0.28 | 0.22 | 0.24 |
| LAI | Soil moisture | -0.14 | 0.18 | 0.46 |
| LAI | Bare-ground cover | 0.11 | 0.11 | 0.35 |
| LAI | Precipitation treatment | 0.02 | 0.16 | 0.89 |
| ANPP | Soil temperature | -0.83 | 0.24 | **0.01** |
| ANPP | Soil moisture | -0.23 | 0.27 | 0.40 |
| ANPP | Bare-ground cover | 0.13 | 0.17 | 0.46 |
| ANPP | Precipitation treatment | 0.24 | 0.24 | 0.33 |
| Bare-ground cover | Soil temperature | -0.02 | 0.27 | 093 |
| Soil temperature | Precipitation treatment | -0.58 | 0.22 | **0.02** |
| Soil moisture | Bare-ground cover | 0.03 | 0.18 | 0.86 |
| Soil moisture | Soil temperature | -0.47 | 0.22 | **0.05** |
| Soil moisture | Precipitation treatment | 0.42 | 0.23 | 0.09 |

**Table S10.** Model estimates, standard errors (SE), and p-values (*P*) from *without hay harvest* under altered precipitation piecewise Structural Equation Model depicting the direct and indirect effects of precipitation treatments and clipping on Focal species height and foliar cover **C_3_ graminoid**. Significant *P* (≤0.05) values are shown in bold. SEM AIC = 150.55 F= 0.55 and *P*-value = 0.76.

| **Response** | **Predictor** | **Estimate** | **SE** | ***P*** |
| --- | --- | --- | --- | --- |
| Focal plant height | Community richness | -0.39 | 0.41 | 0.36 |
| Focal plant height | ANPP | -0.15 | 0.92 | 0.87 |
| Focal plant height | LAI | 0.67 | 0.90 | 0.48 |
| Focal plant height | Soil temperature | 0.57 | 0.78 | 0.48 |
| Focal plant height | Soil moisture | 0.48 | 0.52 | 0.39 |
| Focal plant height | Bare-ground cover | -0.46 | 0.38 | 0.26 |
| Focal plant height | Precipitation treatment | -0.41 | 0.46 | 0.40 |
| Focal plant abundance | Focal plant height | 0.59 | 0.26 | 0.06 |
| Focal plant abundance | Community richness | 0.12 | 0.32 | 0.71 |
| Focal plant abundance | ANPP | 0.26 | 0.68 | 0.71 |
| Focal plant abundance | LAI | 0.02 | 0.69 | 0.98 |
| Focal plant abundance | Soil temperature | -0.45 | 0.60 | 0.48 |
| Focal plant abundance | Soil moisture | -0.67 | 0.41 | 0.15 |
| Focal plant abundance | Bare-ground cover | -0.14 | 0.31 | 0.66 |
| Focal plant abundance | Precipitation treatment | -0.16 | 0.36 | 0.66 |
| Community richness | Soil temperature | 0.15 | 0.39 | 0.71 |
| Community richness | Soil moisture | -0.32 | 0.43 | 0.47 |
| Community richness | Bare-ground cover | -0.30 | 0.27 | 0.30 |
| Community richness | Precipitation treatment | 0.38 | 0.38 | 0.34 |
| LAI | ANPP | 0.75 | 0.19 | **<0.01** |
| LAI | Soil temperature | -0.28 | 0.22 | 0.24 |
| LAI | Soil moisture | -0.14 | 0.18 | 0.46 |
| LAI | Bare-ground cover | 0.11 | 0.11 | 0.35 |
| LAI | Precipitation treatment | 0.02 | 0.16 | 0.89 |
| ANPP | Soil temperature | -0.83 | 0.24 | **0.01** |
| ANPP | Soil moisture | -0.23 | 0.27 | 0.40 |
| ANPP | Bare-ground cover | 0.13 | 0.17 | 0.46 |
| ANPP | Precipitation treatment | 0.24 | 0.24 | 0.33 |
| Bare-ground cover | Soil temperature | -0.27 | 0.26 | 0.30 |
| Soil temperature | Precipitation treatment | -0.58 | 0.22 | **0.02** |
| Soil moisture | Bare-ground cover | 0.03 | 0.18 | 0.86 |
| Soil moisture | Soil temperature | -0.47 | 0.22 | **0.05** |
| Soil moisture | Precipitation treatment | 0.42 | 0.23 | 0.09 |

**Table S11.** Model estimates, standard errors (SE), and p-values (*P*) from *hay harvest* under altered precipitation piecewise Structural Equation Model depicting the direct and indirect effects of precipitation treatments and clipping on Focal species height and foliar cover in **C_4_ graminoids**. Significant *P* (≤0.05) values are shown in bold. SEM AIC = 148.86, F= 0.86 and *P*-value = 0.93.

| **Response** | **Predictor** | **Estimate** | **SE** | ***P*** |
| --- | --- | --- | --- | --- |
| Focal plant height | Community richness | 0.10 | 0.14 | 0.48 |
| Focal plant height | ANPP | 0.09 | 0.28 | 0.76 |
| Focal plant height | LAI | -0.39 | 0.33 | 0.24 |
| Focal plant height | Soil temperature | -0.44 | 0.29 | 0.14 |
| Focal plant height | Soil moisture | -0.26 | 0.18 | 0.16 |
| Focal plant height | Bare-ground cover | -0.28 | 0.13 | **0.04** |
| Focal plant height | Precipitation treatment | -0.03 | 0.18 | 0.86 |
| Focal plant abundance | Focal plant height | 0.60 | 0.15 | **<0.001** |
| Focal plant abundance | Community richness | -0.14 | 0.14 | 0.34 |
| Focal plant abundance | ANPP | 0.25 | 0.29 | 0.38 |
| Focal plant abundance | LAI | -0.07 | 0.33 | 0.84 |
| Focal plant abundance | Soil temperature | 0.12 | 0.30 | 0.69 |
| Focal plant abundance | Soil moisture | 0.09 | 0.19 | 0.63 |
| Focal plant abundance | Bare-ground cover | 0.13 | 0.14 | 0.37 |
| Focal plant abundance | Precipitation treatment | 0.00 | 0.18 | 0.99 |
| Community richness | Soil temperature | 0.24 | 0.21 | 0.26 |
| Community richness | Soil moisture | 0.01 | 0.20 | 0.95 |
| Community richness | Bare-ground cover | 0.10 | 0.14 | 0.50 |
| Community richness | Precipitation treatment | 0.46 | 0.18 | **0.02** |
| LAI | Community richness | 0.13 | 0.06 | **0.05** |
| LAI | ANPP | 0.59 | 0.10 | **<0.001** |
| LAI | Soil temperature | -0.44 | 0.12 | **<0.001** |
| LAI | Soil moisture | -0.11 | 0.08 | 0.18 |
| LAI | Bare-ground cover | -0.07 | 0.06 | 0.24 |
| LAI | Precipitation treatment | -0.03 | 0.08 | 0.74 |
| ANPP | Soil temperature | -0.80 | 0.13 | **<0.001** |
| ANPP | Soil moisture | -0.13 | 0.13 | 0.30 |
| ANPP | Bare-ground cover | -0.11 | 0.09 | 0.24 |
| ANPP | Precipitation treatment | 0.10 | 0.12 | 0.40 |
| Bare-ground cover | Soil temperature | 0.21 | 0.14 | 0.13 |
| Soil temperature | Precipitation treatment | -0.62 | 0.11 | **<0.001** |
| Soil moisture | Bare-ground cover | -0.12 | 0.11 | 0.27 |
| Soil moisture | Soil temperature | -0.52 | 0.13 | **<0.001** |
| Soil moisture | Precipitation treatment | 0.22 | 0.13 | 0.10 |

**Table S12.** Model estimates, standard errors (SE), and p-values (*P*) from *without hay harvest* under altered precipitation piecewise Structural Equation Model depicting the direct and indirect effects of precipitation treatments and clipping on Focal species height and foliar cover **C_4_ graminoids**. Significant *P* (≤0.05) values are shown in bold. SEM AIC = 151.88, F= 5.88 and *P*-value = 0.44.

| **Response** | **Predictor** | **Estimate** | **SE** | ***P*** |
| --- | --- | --- | --- | --- |
| Focal plant height | Community richness | -0.08 | 0.08 | 0.33 |
| Focal plant height | ANPP | -0.02 | 0.11 | 0.88 |
| Focal plant height | LAI | 0.34 | 0.09 | **<0.001** |
| Focal plant height | Soil temperature | 0.19 | 0.11 | 0.09 |
| Focal plant height | Soil moisture | -0.06 | 0.11 | 0.58 |
| Focal plant height | Bare-ground cover | -0.04 | 0.09 | 0.61 |
| Focal plant height | Precipitation treatment | 0.23 | 0.12 | 0.07 |
| Focal plant abundance | Focal plant height | 0.39 | 0.19 | **0.05** |
| Focal plant abundance | Richness | 0.11 | 0.15 | 0.46 |
| Focal plant abundance | ANPP | 0.12 | 0.20 | 0.56 |
| Focal plant abundance | LAI | -0.28 | 0.18 | 0.13 |
| Focal plant abundance | Soil temperature | 0.01 | 0.20 | 0.96 |
| Focal plant abundance | Soil moisture | 0.34 | 0.20 | 0.09 |
| Focal plant abundance | Bare-ground cover | -0.02 | 0.16 | 0.92 |
| Focal plant abundance | Precipitation treatment | -0.24 | 0.23 | 0.29 |
| Community richness | Soil temperature | -0.20 | 0.18 | 0.26 |
| Community richness | Soil moisture | 0.22 | 0.20 | 0.26 |
| Community richness | Bare-ground cover | -0.12 | 0.16 | 0.44 |
| Community richness | Precipitation treatment | -0.05 | 0.22 | 0.84 |
| LAI | ANPP | 0.41 | 0.17 | **0.02** |
| LAI | Soil temperature | -0.25 | 0.17 | 0.16 |
| LAI | Soil moisture | 0.07 | 0.18 | 0.71 |
| LAI | Bare-ground cover | -0.14 | 0.14 | 0.32 |
| LAI | Precipitation treatment | -0.26 | 0.20 | 0.20 |
| ANPP | Soil temperature | -0.44 | 0.14 | **<0.01** |
| ANPP | Soil moisture | 0.23 | 0.16 | 0.16 |
| ANPP | Bare-ground cover | -0.14 | 0.12 | 0.28 |
| ANPP | Precipitation treatment | 0.21 | 0.18 | 0.23 |
| Bare-ground cover | Soil temperature | 0.15 | 0.14 | 0.30 |
| Soil temperature | Precipitation treatment | -0.41 | 0.13 | **<0.01** |
| Soil moisture | Bare-ground cover | -0.28 | 0.11 | **0.02** |
| Soil moisture | Soil temperature | 0.37 | 0.12 | **<0.01** |
| Soil moisture | Precipitation treatment | 0.78 | 0.12 | **<0.001** |

** Figure S1.** Average rainfall (cm) each month for the duration of the experiment (2017). Rainfall data downloaded from https://www.mesonet.org/ and is from Washington county, OK which is closely located at Kessler Atmospheric and Ecological Field Station.

**Figure S2.** *Top left panel*: experimental design showing (top panel) arrangement of precipitation shelters that created the precipitation gradient replicated in three blocks (n=3, N=21): -100%, -80%, -60%, -40%, -20% precipitation reduction, 0% change (no precipitation change), and 50% precipitation addition; and arrows pointing clipped (mimicked hay harvest) and unclipped (no hay harvest) subplots. *Bottom panel shows left*: illustration of a plot and its nested subplots, and *right*: photo of experimental plot. Clipped subplot was clipped once a year during the growing season (clipping treatment), while unclipped subplot was our control.

**Figure S3.** Conceptual a priori model for the precipitation gradient with *hay harvest* and *no hay harvest*. In blue are all effects emerging from the precipitation treatment on focal plant performance (abundance and height), in brown from abiotic variables and green from biotic variables.
